# Identifying Factors Associated with Maternal Deaths in Jharkhand, India: A Verbal Autopsy Study

**DOI:** 10.3329/jhpn.v31i2.16391

**Published:** 2013-06

**Authors:** Nizamuddin Khan, Manas Ranjan Pradhan

**Affiliations:** ^1^Population Council, India Habitat Centre, Lodi Road, New Delhi, India;; ^2^Department of Fertility Studies, International Institute for Population Sciences (IIPS), Mumbai, India

**Keywords:** Delays, treatment, Factors, Maternal death, Verbal autopsy, India

## Abstract

Maternal mortality has been identified as a priority issue in health policy and research in India. The country, with an annual decrease of maternal mortality rate by 4.9% since 1990, now records 63,000 maternal deaths a year. India tops the list of countries with high maternal mortality. Based on a verbal autopsy study of 403 maternal deaths, conducted in 2008, this paper explores the missed opportunities to save maternal lives, besides probing into the socioeconomic factors contributing to maternal deaths in Jharkhand, India. This cross-sectional study was carried out in two phases, and a multistage sampling design was used in selecting deaths for verbal autopsy. Informed consent was taken into consideration before verbal autopsy. The analytical approach includes bivariate analysis using SPSS 15, besides triangulation of qualitative and quantitative findings. Most of the deceased were poor (89%), non-literates (85%), and housewives (74%). Again, 80% died in the community/at home, 28% died during pregnancy while another 26% died during delivery. Any antenatal care was received by merely 28% women, and only 20% of the deliveries were conducted by skilled birth attendants (doctors and midwives). Delays in decision-making, travel, and treatment compounded by ignorance of obstetric complications, inadequate use of maternal healthcare services, poor healthcare infrastructure, and harmful rituals are the major contributing factors of maternal deaths in India.

## INTRODUCTION

Maternal mortality, which reflects the sociocultural and economic disadvantages that women experience, has been identified as a priority issue in health policy and research in India. The Child Survival and Safe Motherhood Programme (1992), Reproductive and Child Health Programme (1997), National Population Policy (2000), and National Rural Health Mission (2005-2012) consistently reiterate the Government's commitment to safe motherhood. India's maternal mortality ratio (MMR) stood at 570 in 1990, which fell to 470 per 100,000 livebirths in 1995, 390 in 2000, 280 in 2005, and 230 in 2008 (1). India, with an annual decrease of MMR by 4.9% since 1990, now records 63,000 maternal deaths a year and tops the list of countries with high maternal mortality. Despite 59% drop in MMR since 1990, the country is far from achieving the national sociodemographic goal of reducing MMR to below 100 by 2010 (2) or to the target of Millennium Development Goal (MDG) of 75% reduction by 2015 (3). Widespread regional variation, besides higher concentration of maternal mortality in specific social groups (religion, caste, or tribe), has also been evident from past studies (4,5,6). Haemorrhage has been found to be the major reason for maternal deaths in India (7,8,9,10,11). Other important determinants found are: sepsis, post-abortion complications, and obstructed labour. Again, delays in decision-making, travel, and treatment independently or in combination contribute to maternal mortality in the country (8).

Jharkhand, the 28^th^ state of India, spreads across 79,714 square km in eastern India and comprises 32,615 villages and 152 towns (12). Out of the 32.9 million population, 76% are rural inhabitants, 28% belong to scheduled tribe (ST), and 12% to scheduled caste (SC) category (13). Female literacy is particularly low in the state (56%), which is again lower in rural areas (50%). Round 3 of the National Family Health Survey (NFHS) reveals that more than half of the households in Jharkhand (52%) fall in the lowest wealth quintile (14) while only one-third of households fall in the top 3 quintiles. The same survey depicted that 63% of the women aged 20-24 years got married before the legal minimum age of 18 years. Total fertility rate was 3.3; among women aged 15-19 years, more than one-quarter (28%) had already begun childbearing. The median interval between two consecutive births was 32 months. Less than one-third (31%) of the currently-married women aged 15-49 years were using any modern contraceptive method while about a quarter (23%) had an unmet need for contraception.

Among mothers who gave birth in the five years preceding the NFHS-3, 57% received antenatal care from a health professional, and only one-third of them received antenatal care during the first trimester of pregnancy. Four out of every five births in Jharkhand took place at home. Only 17% of the mothers had a postnatal check-up within 2 days of childbirth, as is recommended; most women received no postnatal care at all. Among women aged 15-49 years, about two-fifths (43%) were underweight, and 70% were anaemic. Again, merely 12% of the women decided on their own about their healthcare. Above all, the MMR in the state was 312—much higher than the national average of 254 (9). Moreover, higher level of illiteracy, poor economic status, political instability leading to ineffective implementation of policies, poor health infrastructure, and a population that is known for strict adherence to traditional beliefs and practices make the state a perfect place to carry out the study for ascertaining the magnitude and attributes of maternal deaths. Specifically, the objectives were to: (i) understand the missed opportunities to save maternal lives; (ii) explore social dimensions contributing to maternal mortality; and (iii) support the Government and other agencies to develop need-based area-specific strategies to addressing these issues.

## MATERIALS AND METHODS

A cross-sectional study was carried out in 2008 in two phases. Listing of houses in all the villages to enumerate maternal deaths in the study area during the reference period (2004-2007) was followed by selection of a representative sample for verbal autopsy on the maternal deaths that occurred during one year preceding the survey (2006-2007). A multistage sampling design was followed to choose the deaths for verbal autopsy. First, five districts, i.e. Palamau, West Singhbhum, Giridih, Godda, and Gumla, representing five divisions of the state, were selected using simple random sampling. Second, three blocks from each district were selected using systematic random sampling. Third, maternal deaths that occurred during the last one year prior to the study were considered for verbal autopsy. In all, 4,154 maternal deaths were recorded during the reference period (2004-2007) in the five districts. The verbal autopsies on maternal deaths were designed to provide interventions leading to prevent maternal deaths, besides giving an understanding of the causes of maternal deaths. To attain these objectives, a sample-size of 470 out of 883 maternal deaths in the year prior to the survey period (2006-2007) was fixed for verbal autopsies, depending upon time and resource constraints. A separate sample for maternal deaths was drawn using minimum sample-size criteria as well as using survey design effect. Based on 95% confidence interval, proportion 0.5 in the population (for maximum variability), alpha 5% (two-sided), and power of 80%, the minimum sample-size for maternal deaths after applying finite population (total deaths in the last one year), the correction factor comes to be 346, assuming random-sample procedures. Since the sampling design is two-staged, a design effect of 1.25 is assumed and, allowing for a 10% non-response cases for administering the detailed maternal questionnaire, the minimum targeted sample for maternal deaths segment turns out to be 470. Sample allocation was done using the probability proportional to population-size (PPS) methodology from selected blocks of respective districts.

The verbal autopsy tool was designed to collect information from the sample households where maternal deaths occurred. The verbal autopsy tool comprised six stages. The first stage was to collect background information on the deceased. The second stage contained a brief obstetric history of the woman's illness that led to death. In the third stage, general information was collected about events preceding the deaths. Information regarding general illness leading to death was collected, and specific questions on symptoms and signs of the last illness were asked in the fourth stage. The fifth stage covered maternal deaths during pregnancy, delivery, abortion, and within six weeks after delivery or abortion. Information regarding treatment and care-seeking behaviour of the deceased women was also covered in the fifth stage. Additional information on rituals during pregnancy, childbirth, and after delivery performed by the communities was collected in the last stage. Information on maternal deaths was gathered successfully from 417 cases through verbal autopsy technique. Of 417 verbal autopsy cases, 403 were identified as maternal deaths while 14 cases were accidental deaths.

Informed verbal consent from the respondents, i.e. husband or mother-in-law, was obtained before verbal autopsy. The verbal autopsies were conducted by trained research investigators who were post-graduates in social science and received a one-week intensive training to conduct the interviews, including that on ethical issues. The information on causes of deaths was verified by a team of doctors, including one obstetrician, before determining the actual cause of death. The analytical approach includes bivariate analysis through SPSS (version 15), besides triangulation of both qualitative and quantitative findings. Moreover, to derive the MMR, livebirths have been projected from the 2001 Census data for the same periods. It is worth mentioning that the study followed the World Health Organization (WHO) definition of maternal mortality, i.e. the death of a woman while pregnant or within 42 days after termination of pregnancy, irrespective of the duration and site of the pregnancy, from any cause relating to or aggravated by the pregnancy or its management but not from accidental or incidental causes.

## RESULTS

### Maternal mortality ratio

The data in Table 1 show the district-wise and overall MMR (with 95% CI) by time period. The overall MMR in the last three years preceding the survey has reduced from 527 to 376 per 100,000 livebirths. Nevertheless, maternal mortality remains high in the state, which calls for focused attention to overcome this challenge. The reduction in MMR was observed less in Gumla and West Singhbhum compared to other districts over time.

### Context of deceased women

The details about socioeconomic and demographic characteristics of the deceased women have been described in Table 2. The mean age of the deceased women was 27 years, and 85% of them had no education. Majority of the deceased (74%) were housewives, and 16% were working in agricultural sector. On an average, the deceased experienced three pregnancies, and the average parity was 2.8. Deaths of these women left an average of two children motherless at home. Poverty might be one of the leading factors contributing to maternal deaths in the state as most (89%) of the deceased were living in houses made with low-quality material. Moreover, the mean household-size was 6.3, and monthly household income was US$ 35.

Table 3 presents selected characteristics pertaining to maternal healthcare prior to the women's death. Majority (72%) of the deceased did not receive any antenatal care in the pregnancy that ended in their deaths. Only 28% of the deceased received at least one antenatal care visit, which included 7% of cases where the respondents did not remember the number of antenatal care visits. Merely 16% of the deceased had given birth in health facilities. A physician provided care during delivery for only 13% of the cases while, for 6%, a midwife was in charge, and a large majority of the cases (80%) were attended by others (untrained *dai*, relatives, etc.) during birth. Majority of the women (81%) died at home, 12% died in hospital (7.4% in Government and 4.2% in private hospitals), and the rest (7%) died on their way to the hospital/clinic. Data further revealed that 45% of the women died within six weeks after delivery, 28% died during pregnancy, 26% died during delivery, and the rest died within six weeks after abortion.

**Table 1.** Maternal mortality ratios by district, Jharkhand, 2004-2007

District	2004-2005		2005-2006		2006-2007	
	MMR	Lower-upper confidence limits	MMR	Lower-upper confidence limits	MMR	Lower-upper confidence limits
Gumla	597	434-760	537	371-698	474	324-624
West Singhbhum	398	264-533	316	207-424	286	193-379
Giridih	637	468-809	534	408-659	433	319-547
Palamau	635	368-900	521	319-722	444	302-585
Godda	378	249-507	311	200-422	242	151-333
Total	527	448-607	440	374-505	376	322-429

To estimate MMR, livebirths have been projected for the reference period, using the 2001 Census data

**Table 2.** Sociodemographic characteristics of women who died due to maternal causes, Jharkhand, 2006-2007

Characteristics	Statistics (Number)
Age (years): Average, SD	27, 6.8 (403)
Educational level^1^ (%)	
No education	84.9 (342)
Up to primary	5.0 (20)
Up to secondary	5.2 (21)
Up to high school	4.5 (18)
Higher secondary or above	0.5 (2)
Occupation (%)	
Housewifery	74.4 (300)
Working in agriculture sector	16.1 (65)
Working in non-agriculture sector	9.4 (38)
Reproductive history	
Pregnancies (mean, SD)	3.1, 2.1 (403)
Children ever born (mean, SD)	2.8, 2.2 (403)
Children surviving (mean, SD)	1.9, 1.9 (403)
Type of house (%)	
Low-quality material	88.8 (358)
Medium-quality material	8.4 (34)
High-quality material	2.8 (11)
Household monthly income (in Rs)	
Average, SD	1,614, 1,084 (403)
Mean household-size	6.3

^1^Up to primary means 0-5 completed years of schooling; up to secondary means 6-8 completed years of schooling; up to high school means 9-10 completed years of schooling; and higher secondary and above means 11 or more completed years of schooling

### Factors associated with maternal death

Maternal death is mainly attributed to a host of medical factors which are often directly or indirectly influenced by various sociodemographic, economic and cultural issues. The complex nature of maternal complications often misunderstood/ignored due to inadequate knowledge and aggravated by various sociocultural factors in the state is presented in this section.

### A. Deaths immediately after onset of complication

Life-threatening obstetric complications can occur without any warning signs and may immediately lead to maternal deaths.

### Case1: A 26 year-old non-literate scheduled tribe woman with 5 children

The delivery was normal. My daughter-in-law, along with my son, went to her natal family 8 days after delivery. Her family members offered her fish curry which was prepared in their home that day. She did not have the meal and told that she was feeling discomfort in her stomach. Then she complained about headache. She tried to breastfeed her baby but could not. Before her family members could understand anything, she died. This area was very backward dominated by tribal people and hardly received health services provided by the Government.

Her pregnancy was uneventful; so, she and her family did not seek assistance from any health professionals during delivery. She had had five previous births but the family was unaware of the risk of maternal death.

**Table 3.** Reproductive healthcare for women who died of maternal causes, Jharkhand, 2006-2007

Characteristics	Percentage (Number)
Antenatal care for the index pregnancy	
None	72.0 (290)
One	3.0 (12)
Two	9.4 (38)
Three or more	8.4 (34)
Don't know	7.2 (29)
Institutional delivery^*^	16.2 (47)
Personnel at delivery^*^	
Doctor	13.4 (39)
Midwife	6.2 (18)
Others (*dai*, relatives, etc.)	80.4 (234)
Place of death	
Home/community	80.9 (326)
Government hospital	7.4 (30)
Private hospital	4.2 (17)
On the way to hospital	7.4 (30)
Timing of death	
During pregnancy	27.5 (111)
During delivery	25.6 (103)
Within six weeks after abortion	1.5 (6)
Within six weeks after delivery	45.4 (183)

^*^Based on cases who experienced natal care

### B. Delays that resulted in maternal deaths

Verbal autopsy reports of all maternal deaths were examined to illustrate the delays in obtaining appropriate emergency obstetric care. Of the 403 maternal deaths, narratives of family members indicate that, in three-fourths of the deaths, delays were responsible for death. The major types of delays that subsequently resulted in maternal deaths were: delay in decision-making about treatment-seeking after recognition of the complications, delay in arranging means of transport/road connectivity to the health facility, and delay in receiving the actual treatment after reaching the health facility or facilities visited. Table 4 provides information on delays in search for, access to and provision of adequate care.

### Delay in decision-making

The first delay refers to the delay in recognizing emergency obstetric complications and making a prompt decision to seek care. This is particularly important in cases such as postpartum haemorrhage which can lead to maternal death within hours. The data reveal that, in about two-thirds of the cases (65%), it took 2-7 days while, in another quarter of cases, it took more than a week to recognize the complications that led to subsequent deaths. Of those cases where the complications were recognized, only 64% had decided to seek care. Moreover, only 28% of those who had decided to go for treatment had decided to seek treatment from a health facility. The data further reveal that, in almost two-thirds of the cases, the time elapsed between realizing a complication and seeking help was more than one complete day.

### Case 2: A 30-year old non-literate scheduled caste woman with 3 children

My daughter-in-law delivered a live baby. Before delivery, she was not having any complication, except mild fever. The baby died a few hours after delivery as it was delivered preterm, and it was a case of complicated delivery because the foetal position was abnormal in the uterus. The mother was continuously loosing blood and became very weak. The *dai* gave her some traditional roots, besides providing external warmth to the stomach. She died on the 10^th^ day after delivery before we could decide about taking her to a hospital.

**Table 4.** Delays in the search for, access to, and provision of adequate care, Jharkhand, 2006-2007

Characteristics	Percentage (Number)
Delay in decision-making Time required to recognize complication^1^	
≤1 day	5.8 (16)
2-7 days	65.3 (181)
≥8 days	25.3 (70)
Never detected	3.6 (10)
Decision to seek help^1^	64.0 (267)
Decision to seek help in hospital/clinic^2^	28.3 (118)
Time elapsed between realizing that there is a complication and seeking help^1^	
Within one day	34.2 (91)
2-3 days	39.4 (105)
≥4 days	26.5 (73)
Delay in travel	
Time elapsed for arranging the transport^3^	
<1 hour	38.9 (61)
1-<2 hours	30.6 (48)
2-5 hours	22.9 (36)
>5 hours	7.6 (12)
Time elapsed to reach hospital/clinic^3^	
<1 hour	20.4 (32)
1-<2 hours	22.3 (35)
2-5 hours	40.8 (64)
>5 hours	16.6 (26)
Delay in treatment	
Waiting time for care provision^3^	
Immediately/within 30 minutes	46.5 (73)
31-59 minutes	28.0 (44)
<5 hours	17.8 (28)
>5 hours	7.6 (12)
Number of facilities visited^3^	
1	63.1 (99)
2	31.2 (49)
3 or more	5.7 (9)

^1^Based on cases who experienced any pregnancy complication;

^2^Based on cases who sought treatment for any pregnancy complication;

^3^Among those who sought care in hospital/clinic

This case depicts delay in recognizing the obstetric complications, followed by further delay in decision to seek treatment from health facility. Although the woman was suffering from fever before delivery and delivered a preterm baby, she did not have any post-delivery check-up. To make the situation worse, her haemorrhage was treated by an untrained *dai* with traditional herbs that finally led to her death. Institutional delivery or timely treatment by a trained healthcare provider could have saved her from death.

### Delay in travel

The second delay refers to getting to the health facility after the decision is made to seek care. Usually, this is due to the long distance to health facilities, difficulty in getting transport, and associated cost. Majority (70%) of the cases took less than two hours to search for a transport facility while 23% could arrange the transport in 2-5 hours, and 8% took 5 hours or more. Time elapsed to reach hospital/clinic took 2-5 hours in 41% of the cases while 17% took 5 hours or more.

### Case 3: A 30-year old non-literate woman of other backward class (OBC) with 5 children

There was no problem during pregnancy but after eight days of delivery, my wife started complaining of severe stomach-pain. She was unable to urinate from the last night and also had problem in opening the mouth. She became unconscious that night only. We took her to hospital in a bullock-cart the next morning. She died on the way to hospital.

The case shows the poor transportation facility in the state. In spite of delayed decision to seek treatment owing to inadequate awareness of the complications, she could have been saved by reaching the health facility earlier, using any modern means of transport rather than wasting time in the bullock-cart.

### Delay in treatment

The third delay pertains to receiving appropriate treatment when the woman gets to the health facility. Data show that 47% of the women received treatment immediately or within 30 minutes after arrival to the health facility; 28% were treated within 30-59 minutes, and the rest received treatment after a waiting-time of more than an hour. However, some of the respondents perceived that, although women sought and received immediate treatment, the treatment was often substandard in terms of improper identification of the complication and ineffective medicines to address the complication. Arrival at the health facility does not ensure that a woman will receive appropriate treatment to save her life. Data further reveal that 37% of the cases went to a facility where the required care was not available. They then decided to go or were referred to another facility, with substantial delay resulting in death.

### Case 4: A 40-year old non-literate OBC woman with 6 children

She had completed nine months of her pregnancy. When labour-pain started, we took her to the primary health centre (PHC). Doctor treated her with glucose bottle and gave injections. After two hours, the doctor told that the child had died in the womb, and he cannot handle the situation. He suggested taking her to another hospital. Then we proceeded to X city for further treatment but on the way she died. If PHC doctor would have told us earlier, we could save her life in other hospital.

The case reveals the poor preparedness of the PHC to address basic obstetric complications. Timely and appropriate diagnosis and referral by the doctor would have saved her life.

### Multiple delays

More often than not, multiple delays lead to maternal deaths, indicating the need for a properly-functioning health system, along with community awareness to reduce maternal morbidity and mortality. The following case highlights the essence of community awareness about maternal complications.

### Case 5: A 20-year old non-literate scheduled tribe woman in her first pregnancy

She experienced labour-pain at around 4 pm. We called the traditional *dai*. The delivery took place at 7 pm. Both mother and the child were cleaned after placenta came out. During that time only, the woman experienced fits. Her hands and legs were shivering like a ‘chicken’, and her body curved like an ‘arrow’. She even vomited watery things once. Foam started coming out from her mouth. She spent the entire night in that condition and, by morning, she was unconscious. At 7 am in the morning, we took her to Dr. X at place Y in a jeep. As she was still unconscious, rather than giving any oral medicine, she was administered glucose-water. After some time, the doctor told us that it is not possible to treat her at his place and suggested us to take her to the district hospital. Then we took her to her natal home, which was nearby so as to make arrangement for going to that hospital. By then she died.

This case depicts how multiple delays are interconnected. The family members did not feel the need for institutional delivery. There was delay in decision-making to seek treatment during the night when her condition was worsening. The family members took her to a local doctor rather than to a hospital, thus loosing valuable time to save her. Again, the local doctor wasted precious time by attempting to treat her without appropriate expertise to manage the complication. It was compounded further by the delay in arrangement to take her to the hospital as suggested by the local doctor. To avert some of these maternal deaths, creation of awareness among women and their families about maternal complication and its consequences is necessary.

### C. Adherence to traditional rituals

The state is known for its adherence to traditional rituals. However, some of these existing practices lack scientific logic and often adversely affect the maternal health. Findings suggest that many a time women are discouraged to have adequate food during pregnancy, enhancing the risk of anaemia and associated maternal complications. Though not encouraged, sometimes pregnant women consume locally-available alcohol to avoid any pain. Again, delivering the baby with the help of relatives or at most by a traditional *dai* is quite common. Moreover, *dai's* treatment is often sought in case of any post-delivery complication as well.

### Case 6: A 26-year old non-literate scheduled tribe woman with 5 children

In our community, we generally discourage pregnant women to have much food. Slight amount of country-made liquor (*Handia*) is given to pregnant women in case of pain.

### Case 7: A 37-year old non-literate OBC woman with 6 children

When my daughter-in-law was in her 6^th^ month of pregnancy, she went to her natal home to have *Prasad* (food items consumed after dedicating it to god, which may be fruits or cooked grains/cereals). After returning, she started behaving abnormally and complained of seeing some evil spirit. We called the priest (*Ojha*) from the neighbouring village to protect her from any evil spirit. She was normal for some days and then again complained of headache and non-interest in food. The local doctor who visited her told that she was anaemic and gave her some medicines. During delivery when she experienced more pain, the *dai* was called in. The *Dai* told that the baby was in a wrong position and gave massage in her stomach to deliver the baby. The day after delivery, she felt breathlessness, along with severe body-pain. The same local doctor was called in, and he said that the lady was having stomach-pain due to massage during delivery and gave some medicines but the pain continued, and she died that day. Colour of her urine was like that of mustard oil by that time

### D. Unsafe abortion

Unsafe abortion has been found to be an important determinant of maternal death. The study revealed that many people were not aware of abortion being legal in the country. Again, many were not only unaware of the consequences of unsafe abortion but also lacked knowledge about the facilities providing safe abortion. The following case shows the ignorance that led to unsafe abortion attempt, resulting in maternal death.

### Case 8: A 25-year old non-literate OBC woman with 1 child

As I (husband) was not interested for another baby in a short interval, I brought some medicines from the pharmacist and fed her (to abort the child) during second month of her pregnancy. A week after taking the medicine, one day after taking usual bath, she told that she was feeling uneasy and lied down. When her condition started worsening, we took her to X sadar hospital. By that time, she had lost a lot of blood. She died in between treatment.

### E. Poverty

Poverty is widespread in the state, and many a times, hinders the treatment-seeking for any maternal health complications. The following case reveals the plight of a husband who could not seek proper treatment for her wife due to financial constraints.

### Case 9: A 25-year old non-literate scheduled tribe woman with 8 children

After delivery, she suffered itching in the whole body. When she was pregnant, she was suffering from fever, along with cough and cold. She had night-blindness during pregnancy. Moreover, there was scarcity of food in our home. We did not have money for her treatment; still then I took some loan from the money-lender (*Mahajon*) and treated her in the nearby facility of a local doctor (*Jhola Chhap doctor*). I could not think of taking her to a better hospital outside due to financial problem, and she died.

### F. Poor health infrastructure

The respondents at large perceive that the available health facilities are inadequate, inaccessible, without required staff and, above all, are providing poor-quality services.

### Case 10: A 30-year old non-literate scheduled tribe woman with 5 children

When she was in her third month of pregnancy, she suffered from cold and fever. We treated her by the local village doctor (*Jhola Chhap doctor*) for 15 days but in vain. She became very weak, and we admitted her to X hospital. The doctor started the treatment but could not cure her fever. Her condition did not improve even after spending 15 days in the hospital, and she died in the hospital.

The above case shows the possible inability of the village doctor to diagnose the real disease that could have saved one precious life.

## DISCUSSION

Medical and socioeconomic factors leading to maternal mortality are largely preventable. Nevertheless, it remains a major public-health challenge in the state of Jharkhand, India. The MMR in the state is much higher than the national figure, questioning the implementation of the National Rural Health Mission (NRHM); the state needs specific policies/programmes, targeting maternal health. The study finds that most of the deceased were poor; non-literate, and housewives, indicating that certain sections of the society are more prone to maternal mortality. This validates the results from past studies (4,5,6,7). Reducing maternal mortality in the state would entail a multipronged approach. Provision of quality obstetric care, besides improving health infrastructure (such as human resources) and capacity-building of the existing health workforce, seems pertinent. Moreover, behaviour change communication programmes targeting community mobilization to increase the use of obstetric care and programmes addressing awareness-generation toward maternal complications are equally important.

### Provision of quality obstetric care

The study reveals the fact that the use of maternal healthcare services is remarkably low in the state, and that often leads to maternal complications and subsequent deaths. The verbal-autopsy sample showed that 7 of every 10 deceased women did not receive any antenatal care that resulted in their deaths. The poor use of antenatal care services was also indicated by a series of NFHSs conducted in 1998-1999 and 2005-2006 in the state (Annexure). During the seven years between NFHS-2 and NFHS-3, the number of women with at least three antenatal care visits has increased by 47%, and those receiving antenatal care within the first trimester of pregnancy have increased by 87%. However, the percentage of women receiving the aforesaid services is still below 40%.

Less than one-fifth of the deliveries were institutional. Moreover, only a 5 percentage-point increase in institutional delivery (from 14% to 19%) in seven years indicates that the performance of reproductive and child health programmes in the state was not satisfactory. Additionally, only 29% of the deliveries were attended by health professionals, which was again lower in rural areas (21%) compared to urban areas (62%). Poor institutional deliveries have also been revealed from verbal-autopsy sample as only 17% of the deceased gave birth in a health facility, and merely 20% of the deliveries were assisted by health professionals. This may be because of inadequate availability and accessibility to the existing healthcare facilities and/or services. Other probable factors were the shortfall in both allotted and filled-in positions of gynaecologists/obstetricians and nurse-midwives in the state.

The current use of any family planning method during the study period in the state was only 36%, albeit showing an increase of 29% during 1998-1999 to 2005-2006. Nevertheless, unmet need for family planning has increased by two percentage-points during the same period and, at present, 23% women have an unmet need for family planning. Poor performance of family planning programme may have led to unintended births, which might, in turn, have led to maternal deaths in the state. Further, within the state itself, the health indicators of groups which face social exclusion and denial of rights and entitlement on account of caste, class, and sex were poorer. Social exclusion was especially high for SC/ST groups, leading to overall poor health indicators. Poor awareness and use of the maternal healthcare services urge the need for reinforcement of home-visits by the grassroots-level healthcare providers.

Antenatal care presents an opportunity for early detection and treatment of anaemia in pregnancy. Additionally, culturally-appropriate nutritional counseling should be provided during antenatal care. Adequate training of physicians and equipping all PHCs, First Referral Units (FRUs), and district hospitals in the provision of comprehensive obstetric care would address the high burden of obstetric complications. Gynaecologists/obstetricians should be deployed round the clock in all Community Health Centres (CHCs). Obstetric record-keeping should be improved to monitor the performance of health facilities.

### Health systems strengthening and capacity-building of health workforce

As reported by the respondents and corroborated by the available government statistics, the healthcare infrastructure, especially associated with maternal healthcare, was far from satisfactory. There were 194 CHCs, 321 PHCs, and 3947 subcentres functioning in the state (12). The PHCs which provide the basic emergency obstetric care in the community had a shortfall of 60% (number of PHCs functioning against required number). To make the situation worse, there was a 22% shortfall in the required number of subcentres which, along with the provision of basic maternal healthcare services, works as a catalyst in terms of awareness-generation through home-visits by auxiliary nurse-midwives (ANMs). Additionally, a shortfall of health specialists in the existing health facilities was another problem in the state. There was a 91% shortfall in the number of obstetricians and gynaecologists at CHCs, and a 74% shortfall in the number of nurse-midwives/staff nurses at PHCs and CHCs. Again, only 44% of the subcentres were functioning in a government building, and merely 15% of them have ANM quarter, thus further inhibiting the access to the provision of services. Additionally, an assessment of the round-the-clock health facilities in the state reveals that none of the CHCs, Block PHCs (PHCs providing round-the-clock services at block headquarter), and PHCs was equipped with round-the-clock emergency services (15). Moreover, verbal autopsies showed that the private hospitals and private nursing homes were more capable of providing emergency obstetric care compared to the government health facilities. The above-discussed figures not only suggest the need for health systems strengthening to deliver high-quality services, particularly in the underserved communities, but also for health workforce capacity-building by implementing national plans to train, retain, and deploy health workers. Public-private partnership in service delivery, especially in hard-to-reach areas, would yield dividends in terms of higher service-use. Moreover, untrained *dais* should be trained to recognize the obstetric complications at an early stage and refer the high-risk cases for adequate management.

### Awareness-generation to avoid the delays in decision, travel, and treatment

The delays that have been found to be the major reasons for maternal mortality in the state may be attributed to many factors; inadequate awareness of maternal complications being the main. Information, education and communication (IEC) activities targeting behaviour change in the community toward the importance of maternal health and negative consequences of traditional practices that unfavourably affect maternal health, would definitely work toward saving precious maternal lives.

### Community mobilization to use obstetric care

Women dying because of pregnancy or delivery complications mostly in their homes reveal the poor quality of delivery and emergency obstetric care services in the community, besides inadequate awareness of complications. This poor awareness and use of services, and their correlates urge multifaceted communication campaigns to encourage delivery by skilled birth attendants. Educating women and their families about the benefits of delivery by skilled birth attendants and encouraging family preparedness in terms of arrangement of money, accompanying persons, and transport facility seem pertinent. Awareness of the financial benefits to mothers delivering in an institution through the *Janani Suraksha Yojana* (JSY) scheme under the National Rural Health Mission would further enhance institutional deliveries, along with reducing the delay in decision-making for some families. Efforts to strengthen the home-visits by the existing grassroots-level health workers, such as ANMs or Accredited Social Health Activists (ASHAs) would certainly be helpful in this regard. Moreover, introduction of emergency transport services (proven to be successful in the state of Andhra Pradesh) would undoubtedly diminish the delay in transportation. Renewed and re-invigorating efforts to eliminate these preventable causes of maternal mortality are important for the betterment of the society in general and women in particular.

## ACKNOWLEDGEMENTS

The research study was part of a three-year project titled “The Innovations in Family Planning Services-Technical Assistance Project” (ITAP) funded by United States Agency for International Development (USAID) and implemented by the Futures Group International India Pvt. Ltd. The authors are thankful to the Futures Group International India Pvt. Ltd. for providing the opportunity to write this paper.

**Annexure.** Changes in maternal health indicators between 1998-1999 and 2005-2006

Indicator	1998-1999	2005-2006	% change
Women with no education	61.4	58.5	4.7 (-)
Women having any antenatal care (%)	42.0	60.7	44.5 (+)
Women having at least three antenatal care visits (%)	24.5	36.1	47.3 (+)
Women having antenatal care within the first trimester of pregnancy (%)	17.4	32.5	86.8 (+)
Presence of skilled attendant at delivery (%)	17.6	29.1	65.3 (+)
Number of institutional deliveries (%)	14.0	19.2	37.1 (+)
Contraceptive prevalence (%)	27.6	35.7	29.3 (+)
Unmet need for family planning	21.0	23.1	10.0 (+)

Source: National Family Health Survey 2 and 3; (+)=Increased; (-)=Decreased
